# Evidence of Antimicrobial Resistance in Bats and Its Planetary Health Impact for Surveillance of Zoonotic Spillover Events: A Scoping Review

**DOI:** 10.3390/ijerph20010243

**Published:** 2022-12-23

**Authors:** Popy Devnath, Nabil Karah, Jay P. Graham, Elizabeth S. Rose, Muhammad Asaduzzaman

**Affiliations:** 1 College of Veterinary Medicine, Washington State University, Pullman, WA 99164, USA; 2 Department of Microbiology, Noakhali Science and Technology University, Noakhali 3814, Bangladesh; 3 Department of Molecular Biology and Umeå Centre for Microbial Research, Umeå University, SE-901 87 Umeå, Sweden; 4 School of Public Health, University of California, Berkeley, CA 94720, USA; 5 Vanderbilt Institute for Global Health, Vanderbilt University Medical Center, Nashville, TN 37203, USA; 6 Department of Community Medicine and Global Health, Institute of Health and Society, Faculty of Medicine, University of Oslo, 450 Oslo, Norway; 7 Planetary Health Alliance, Boston, MA 02115, USA; 8 Planetary Health Working Group, Be-Cause Health, Institute of Tropical Medicine, Nationalestraat 155, 2000 Antwerp, Belgium

**Keywords:** antimicrobial resistance (AMR), bats, zoonotic spillover, planetary health, one health

## Abstract

As a result of the COVID-19 pandemic, as well as other outbreaks, such as SARS and Ebola, bats are recognized as a critical species for mediating zoonotic infectious disease spillover events. While there is a growing concern of increased antimicrobial resistance (AMR) globally during this pandemic, knowledge of AMR circulating between bats and humans is limited. In this paper, we have reviewed the evidence of AMR in bats and discussed the planetary health aspect of AMR to elucidate how this is associated with the emergence, spread, and persistence of AMR at the human–animal interface. The presence of clinically significant resistant bacteria in bats and wildlife has important implications for zoonotic pandemic surveillance, disease transmission, and treatment modalities. We searched MEDLINE through PubMed and Google Scholar to retrieve relevant studies (*n* = 38) that provided data on resistant bacteria in bats prior to 30 September 2022. There is substantial variability in the results from studies measuring the prevalence of AMR based on geographic location, bat types, and time. We found all major groups of Gram-positive and Gram-negative bacteria in bats, which are resistant to commonly used antibiotics. The most alarming issue is that recent studies have increasingly identified clinically significant multi-drug resistant bacteria such as Methicillin Resistant Staphylococcus aureus (MRSA), ESBL producing, and Colistin resistant Enterobacterales in samples from bats. This evidence of superbugs abundant in both humans and wild mammals, such as bats, could facilitate a greater understanding of which specific pathways of exposure should be targeted. We believe that these data will also facilitate future pandemic preparedness as well as global AMR containment during pandemic events and beyond.

## 1. Introduction

Antimicrobial resistance (AMR) is a global One Health (OH) issue that involves various species, including wildlife, and containment requires a holistic approach. While drug-resistant pathogens are causing a high disease burden in terms of disability-adjusted life-years and substantial economic loss to the public health sector [1,2], the role of the environment and spillover from wild animal reservoirs needs more attention [3]. There is increasing evidence of the spread of pathogenic drug-resistant bacteria in wild animal populations, including wild mammals [4,5,6]. Bacterial antimicrobial profiling of wildlife, including those found in both wild and urban environments, are crucial to OH prevention strategy development for AMR. Excessive and inappropriate use of antibiotics in human and animal health as well as agricultural farming practices has led to the huge rise in AMR around the world [7,8]. Frequent AMR reports in animal, wildlife, and environmental samples demonstrate its massive proliferation and have contributed to the subsequent spread of resistance in humans [9]. Wildlife is reported to be a reservoir of several bacterial pathogens with high levels of AMR and is a vector for spreading bacterial zoonoses to humans [10]. Several recent studies reporting carbapenemase [11,12] and ESBL-producing bacteria in wildlife [13,14] raise a major concern for further investigating the AMR issue in both wildlife and domestic animal origin. Due to increased interaction between wildlife and humans, it is clinically important to have a clear understanding of the AMR profile of wildlife [15]. Yet, the rates, modes, and drivers of acquisition are unclear, under-investigated, or inadequately reported [16].

Major infectious diseases causing epidemics and pandemics have emerged as zoonoses [17]. Most of the zoonotic pandemics, such as avian flu, swine flu, severe acute respiratory syndrome (SARS), Middle East respiratory syndrome (MERS), Ebola, Zika, Nipah, and Henipavirus viral diseases, have been recognized as a global public health emergency for many years due to their rapid spread and extensive virulence [18,19,20,21]. The frequent emergence of zoonotic epidemics or pandemics and the use of antimicrobial agents to control secondary infections also triggers the rise of AMR [22,23]. Obviously, the thousands of species of wild animals are likely to be an important source of animal-to-human spillover of zoonotic pathogens [24]. Notably, most of these zoonoses (e.g., SARS, MERS, Ebola, Nipah encephalitis, and corona virus) are transmitted from bats to humans [25,26] directly or through intermediate hosts [19,27,28,29]. In addition, bats are one of the potential vectors for the transmission of both viral and bacterial zoonotic pathogens [30,31]. Considering the role of bats in most recent pandemics and disease spillover [24], the presence of drug resistant pathogens in bats and their likely impact on the global AMR burden needs to be studied.

Bats are one of the free roaming wild mammals belonging to the order Chiroptera, a diverse group with a specific life cycle and different feeding habits [32]. With more than 1300 species, bats are one of the most diverse classes of mammals, representing approximately 20% of the world’s mammal population [32,33]. On a worldwide scale, approximately half of all known bat species spend part of their lives in caves, with 32% indigenous to a single nation and 15% currently threatened [34,35]. This review has reported common bat species containing AMR genes and bacteria, indicating anthropogenic contamination. To understand what causes those bats to have such an AMR profile, we must first understand their environment, roosting habits, and feeding habits.

Myotis species were found to be the most prevalent bat species in this review. These bats are insectivorous and feed mostly in complex habitats (i.e., cluttered) [36,37]. Many of them were discovered near open spaces and vegetation along water bodies [37]. Interestingly, Myotis species have been discovered hibernating in caves with a wide range of microclimate conditions, whereas their breeding populations have primarily been established in buildings [37,38,39]. Another widespread species was the straw-colored fruit bat (*Eidolon helvum*), a huge Old World fruit bat (Pteropodidae) found across sub-Saharan Africa [40,41]. It feeds upon a large variety of pollen, fruit, leaves, and bark of native plants [42]. This species roost in enormous numbers on trees, reaching hundreds of thousands of individuals. Roosts are frequently found in metropolitan settings, leaving them especially sensitive to human interactions [41].

Flying foxes (*Pteropus* spp.) display great coloniality and build enormous colonies of hundreds to millions of individuals in a typical roosting place known as a camp [43]. Because of fruiting phenology, many frugivores, such as flying foxes, exhibit seasonal dependence in food distribution and availability [43,44].

The bat species known as the vampire bat (*Desmodus rotundus*), a sanguivorous (blood-eating) wild mammal was also widespread, found in the Americas from northern Mexico to central Chile and Argentina [45]. *D. rotundus* is one of three mammal species that depend only on blood, primarily from domestic animals, but larger wildlife and humans also may serve as food sources on occasion [45]. The feeding habit of vampire bats enhances their ability to transmit pathogenic microorganisms [46].

Anthropogenic activities to meet human needs have already affected at least 70% of terrestrial ecosystems, and such disruption can cause changes in animal activity patterns, energy expenditure, physiological features, foraging behavior, reproductive success, and roosting behavior [41,47]. Bats often range from deep forests to densely populated localities. Consequently, they acquire a wide variety of microorganisms, ranging from deadly viruses to multidrug resistant (MDR) bacterial pathogens. Despite bats being a potential reservoir of bacterial pathogens, extensive studies in the search for bat bacterial flora are lacking [32,48]. Bacterial isolates from bats can acquire high levels of resistance from human and domestic animals, or vice versa, through contact as per previous reports [30].

In this paper, we have reviewed the published literature focusing on AMR in bacterial isolates from bats to elucidate its association with the emergence and spread of global AMR. Our study findings will help the development of OH policies and initiatives for reducing the spread of AMR from wildlife, particularly in the time of zoonotic pandemics.

## 2. Materials and Methods

To find relevant literature addressing AMR in bats, we searched two bibliographic databases. MEDLINE through PubMed and Google Scholar databases were searched using the key word “bats” and different combinations of the following terms: antimicrobial resistant, antimicrobial resistance, antimicrobial susceptible, antimicrobial susceptibility, antibiotic resistant, antibiotic resistance, antibiotic susceptible, antibiotic susceptibility, antimicrobial susceptibility, multidrug resistant, and multidrug resistance. We only considered peer-reviewed articles authored in English and released prior to 30 September 2022. The retrieved publications were screened using the Rayyan QCRI systematic review program [49] and were independently evaluated for inclusion by two review authors. When conducting the full-text screening, we explained the reasons why certain publications were excluded. Discussion with a third review author helped to settle any disagreements amongst the independent review authors. One review author (P.D.) extracted the data, while another author crosschecked it (M.A.). Disagreements were resolved through team discussion. An accurate visual summary of the screening procedure has been provided using the PRISMA (Preferred Reporting Items for Systematic Reviews and Meta-Analyses) flow diagram [50]. Supplementary Table S1 provides a summary of all included papers (*n* = 38) (Table S1). The inclusion and exclusion criteria of these articles are described below.

Inclusion criteria:

Studies published in English on the prevalence of AMR in bacterial isolates from bats focused on the following issues.

- Bacterial pathogens;
- Bat specimens such as feces, skin swab, oral, and rectal swab/cloacal swabs, etc.;
- Drug sensitivity testing done in a laboratory setting with/without Clinical and Laboratory Standards Institute (CLSI) and/or other standard organizations cutoffs for drug susceptibility testing;
- Reports of resistance genes and plasmids in isolated bacterial samples.

Exclusion criteria:

Duplicated population groups, editorials, perspectives, intervention studies, experimental studies, and narrative reviews studies with inadequate data including;

- Review articles;
- Studies on bacteria isolated from bats without antimicrobial susceptibility, gene, or plasmid detection test results;
- Studies that did not specify bacterial antimicrobial susceptibility isolated from bats.

## 3. Results

**Figure 1:**
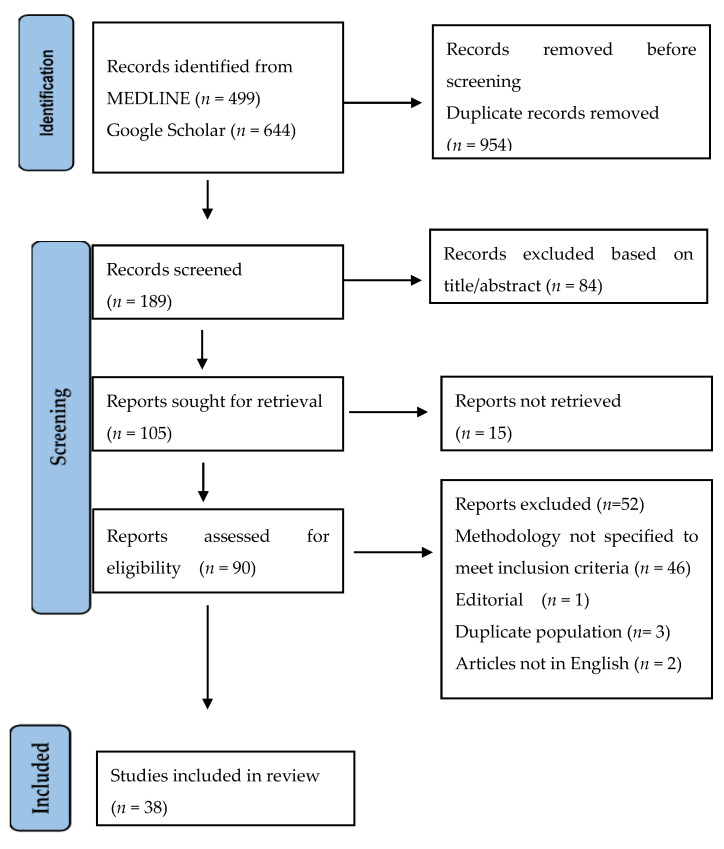
PRISMA flow diagram.

Our initial search identified 1143 records in total. Following screening for duplicates and eligibility criteria, 38 papers were pertinent to the topic of AMR in bats. Finally, we considered data extraction for these 38 papers after the full-text evaluation (Figure 1).

**Figure 2:**
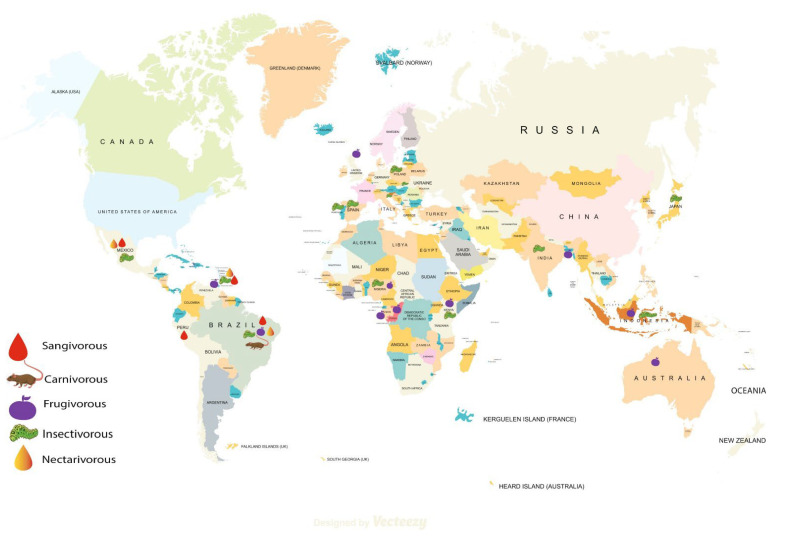
Geographic distribution of bat species in the included studies.

For better reporting of our reviewed articles, we have mapped the bat species (Figure 2) and have categorized the studies based on the bacterial isolates—Gram-negative and Gram-positive. Within Gram-negative bacterial pathogens, we found *Escherichia coli* (*E. coli*) as the most frequently studied and reported bacteria followed by *Enterobacter* spp., *Salmonella* spp., and *Klebsiella* spp. Similarly, *Staphylococcus aureus* and other *Staphylococcus* spp. were the most common Gram-positive bacteria in bats. We described all studies by geographical region since understanding of AMR among isolates in bats might help to understand the history in specific regions and to predict the geographical spread of AMR and epidemics later.

### 3.1. Evidence of Antibiotic Resistant Gram-Negative Bacterial Pathogens in Bats

Gram-negative bacteria constitute the major share of the WHO priority list of drug resistant bacteria with public health importance [51]. Undoubtedly, effective interventions to halt their spillover from wildlife to humans is crucial and need to be implemented. While acquisition of AMR in bacteria isolated from several bat species have been reported in many countries across the world [52,53], we have presented all Gram-negative AMR bacteria based on species and geography to better understand the policy implications.

#### 3.1.1. *Escherichia coli* (*E. coli*)

In 1988, an Indonesian study was the first to reveal the AMR patterns of enteric bacteria isolated from bat feces and reported isolation of *E. coli* (*n* = 15), resistant to sulphamethoxazole (27%), cephalothin (20%), and trimethoprim (7%) [54]. Later in 2005, a Malaysian study also reported isolation of *E. coli* from bats with low or no resistance to treated antibiotics except carbenicillin and streptomycin (7.7% each) [55]. Additionally, a study from Japan (2014) reported *E. coli* (*n* = 26) isolated from bats and found no resistance towards most antibiotics, such as ampicillin, chloramphenicol, and nalidixic acid except streptomycin and chlorotetracylcine (3.8%) [56].

Moving from Asia to sub-Saharan Africa, we found several studies in this region focusing on pathogenic Gram-negative bacteria from different species of bats [57,58,59,60]. Three Nigerian studies reported AMR patterns of bat *E. coli* and high levels of cephalosporin resistance [59,61]. One study reported more than 80% of isolates resistant to cefuroxime, ceftazidime, and ceftotaxime [59], while another study of *E. coli* (*n* = 35) reported low cephalosporin resistance [62]. The most recent study, by Oladiran et al., from Nigeria reported 83.3% of the isolates showing resistance towards augmentin [61]. These isolates were mostly resistant to ampicillin (48%) and tetracycline (37%) [62]. A Kenyan study found ampicillin, streptomycin, and trimethoprim resistance among isolates [60]. Similar to the Nigerian and Kenyan studies, a report from Gabon also stated high levels of cephalosporin and beta lactam resistance in bat *E. coli* (*n* = 6) [33]. All isolates were resistant against amoxicillin, ampicillin, ticarcillin, cefotaxime, ceftazidime, cefpodoxime, aztreonam, cephalexin, erythromycin, and streptomycin [33]. Notably, more than 80% of the isolates were also resistant to piperacillin, ciprofloxacin, and trimethoprim [33]. However, a study from the Democratic Republic of bat *E. coli* isolates to antibiotics, except relatively high resistance to Doxycycline (89%) [58].

We found that more recent studies were conducted in Europe, and molecular identification of AMR genes were also reported from some studies [31,52,63,64,65,66]. However, two studies from Portugal using *E. coli* (*n* = 19 and 42) isolates reported very low resistance to amoxicillin, sulfamethoxazole-trimethoprim, and tetracycline and high resistance to cefotaxime and ampicillin [63,66]. Ampicillin resistance among *E. coli* also were found in a Polish study [31]. Similar to previous studies with high streptomycin resistance [33,67], this study reported high Kanamycin resistance (84%), another aminoglycoside.

In the Americas, two studies from Brazil observed diverse species of bats and isolated hundreds of *E. coli* species from bats’ fecal and oral samples [32,48]. Both studies reported low levels of resistance towards antibiotics such as amoxicillin-clavulanic acid, gentamicin, and imipenem. Against other antibiotics, the sensitivity rate was higher except ampicillin (57%) and amoxicillin (54%). A third Brazilian study also reported *E. coli* (*n* = 17) isolates from bats resistant to ampicillin (59%) and amoxicillin (35%) [48]. Two back-to-back reports from Peru reported ESBL-producing *E. coli* (*n* = 5 and 18) from bats which showed pan-resistant to amoxicillin, amoxicillin ticarcillin, piperacillin, cefotaxime, and other antibiotics [52,68]. A study from Trinidad reported isolation of *E. coli* (*n* = 49) from several bat species and found most of them were resistant to erythromycin (71%) and streptomycin (26%) [67]. In a 1999 study, *E. coli* were obtained from a broad variety of mammalian species samples with Australian and Mexican origins [69]. Among the Mexican isolates, a much higher frequency of antibiotic resistance was detected among the bats’ isolates than those obtained from other wild mammals. These isolates demonstrated resistance to streptomycin (100%), ampicillin (46%), and neomycin (15%). In another Australian study, high ampicillin (100%), tetracycline (69.2%), and sulfamethoxazole-trimethoprim (30.7%) resistance in beta-lactam resistant *E. coli* from bats was observed [70]. The detailed drug resistance profile of *E. coli* isolates from bats around the globe has been provided in the Supplementary Table S2 (Table S2).

#### 3.1.2. *Enterobacter* spp.

Similarly, the Indonesian study, mentioned earlier, reported isolation of *Enterobacter* (*n* = 24) from bats that were resistant to cephalothin (96%), ampicillin (67%), and tetracycline (50%) [54]. Later in 2018, a Brazilian study reported isolating *Enterobacter* (*n* = 20) and all isolates showed low resistance to all classes of antibiotics except ampicillin and amoxicillin (>80% isolates were resistant) [48]. A 2020 study from Gabon, however, reported that all the *Enterobacter* isolates were resistant amoxicillin, ampicillin, amoxicillin-clavulanic acid, aztreonam, cefotaxime, cefepime, ceftazidime, and many other antibiotics [33].

#### 3.1.3. *Salmonella* spp.

Reports of *Salmonella* spp. were quite low, but studies were identified in Bangladesh [71], Australia [72], Trinidad [67], and Brazil [32]. In 2009, *Salmonella* spp. from bats were reported from Trinidad that were highly resistant to streptomycin (100%) and erythromycin (75%) [67]. From Bangladesh and Australia, bats’ fecal specimens were reported with *Salmonella serotype Virchow* and *Salmonella Typhimurium* ST19, respectively, and the isolates showed no resistance to any antibiotics [71,72]. Additionally, isolation of *Salmonella* spp. resistant to ampicillin (50%) and cephalexin (50%) were reported from Brazil [32].

#### 3.1.4. *Klebsiella* spp.

An Indonesian study also reported isolation of *Klebsiella* spp. from bats’ fecal samples in 1988 [54]. *Klebsiella* (*n* = 11) isolates were found showing high resistance towards ampicillin (82%) and sulphamethoxazole (27%). A study from Japan noted isolation of *K. pneumoniae* (*n* = 38) from bats but reported only sulfadimethoxin resistance (13%) [56]. Isolation of *K. oxytoca* (*n* = 13) showing high ampicillin resistance (61.5%) also were reported from Brazil [32]. In 2020, *Klebsiella* spp. were isolated in a study from Gabon but the study represented only 4 isolate antibiograms. Interestingly, all these isolates were resistant to 18 types of antibiotics including ampicillin, amoxicillin, kanamycin, nalidixic acid, ceftazidime, cefotaxime, and others [33]. Over the past three decades, the multidrug resistant (MDR) and hypervirulent *K. pneumoniae* lineages have increased. An Australian study investigated the occurrence of *K. pneumoniae* species complex (KpSC) in fruit bats and found none belonged to the MDR clonal lineages that cause frequent nosocomial outbreaks and no isolates were characterized as hypervirulent [53]. All the isolates were resistant to ampicillin and amoxicillin-clavulanic acid.

#### 3.1.5. ESBL-Producing and Colistin Resistant *Enterobacterales*

Drug resistance by ESBL-producing Enterobacterales has been drastically increasing in animals and humans [63,73,74]. This increase has been caused mainly due to acquiring ESBL producing genes by this order. Among the many ESBLs described in a variety of pathogens, *CTX-M*, *TEM*, and *SHV* types proved to be the most predominately detected across the world in animals and humans [75,76,77]. Other than ESBL-producers, colistin resistant Enterobacterales are also a global health threat. Despite having neurotoxic and nephrotoxic side-effects [78], colistin has been reintroduced as a final therapeutic choice for the treatment of carbapenem-resistant Gram-negative infections [79].

While ESBL genes are commonly detected in Gram-negative pathogens isolated from animal origin [80], their presence in bats with different feeding habits, such as sangivorous, insectivorous, and frugivorous, also were frequently observed [52,63]. Benavides et al. first reported the presence of ESBL-producing *E. coli* in vampire bats (*D. rotundus*) in Peru, suggesting a wide dissemination of AMR bacteria in the community [52]. All the 5 ESBL-producing *E. coli* isolates expressed plasmid *bla*_CTX-M-15_ genes showing resistance towards β-lactam antibiotics. Two years later, the authors reported isolation of several genes, such as *bla*_CTX-M-15_ (39%), bla_CTX-M-3_ (11.1%), *bla*_CTX-M-55_ (44.4%), *bla*_CTX-M-65_ (5.5%), bla_TEM-1B-like_ (66.7%), and *bla*_TEM-176_ (28%), responsible for ESBL production [68].

The study from Gabon reported multi-resistant ESBL-producing Enterobacteriaceae with 11 ESBL-producing bacterial isolates (*E. coli* = 6; *K. pneumoniae* = 4; *E. cloacae* = 1) from fruit bats that carried *bla*_CTX-M-15_ and *bla*_SHV-11_ as the ESBL-producing genes [33]. The isolation of ESBL-producing *E. coli* from European free-tailed bats (*T. teniotis*) was first reported in Portugal [66]. The more prevalent beta-lactamase genes detected were *bla*_CTX-M-1_ (57.9%) and *bla*_CTX-M-3_ (36.8%), followed by *bla*_SHV_ (31.6%), *bla*_TEM_ (21.1%), *bla*_OXA_ (10.5%), and *bla*_CTX-M-9_ (10.5%). Presence of *CTX-M* and *TEM* groups in two *E. coli* confirmed the presence of ESBL genes encoding the enzymes in a study in Poland [31]. The sequencing confirmed that these genes were *bla*_CTX-M-3_, *bla*_CTX-M-15_, and *bla*_TEM-1_. Later, a study from Australia also reported high bla_TEM_ gene (92%) acquisition by beta lactam resistant *E. coli* with the detection of *bla*_CTX-M-27_ (7.6%) in low levels [70]. McDougall also reported *K. pneumoniae* isolates having high *bla*_SHV-110_, which is also responsible for beta lactam resistance [53]. From both studies in Australia, detection of *bla*_OXA-1_ (22.22%) in ESBL-producing *E. coli* isolates [70] and *bla*_okpc-1_ (20.5%) in *Klebsiella* isolates from bats were reported [53]. In Algeria, carbapenemase producing genes *bla*_OXA-48_ gene and *bla*_KPC-3_ in two carbapenemase producing *K. pneumoniae* isolate were reported [5].

A study conducted in Gabon [33] found 54.5% of 11 carbapenem resistant isolates to be colistin resistant and the resistant bacteria included *E. cloacae*, *E. coli,* and *K. pneumoniae* (4/6). Similarly, a study from Poland [31] also reported 7.9% colistin resistant *E. coli* out of 38 isolates. However, no studies described any molecular detection of colistin resistance genes from bats such as mobilized colistin resistance (MCR).

**Figure 3:**
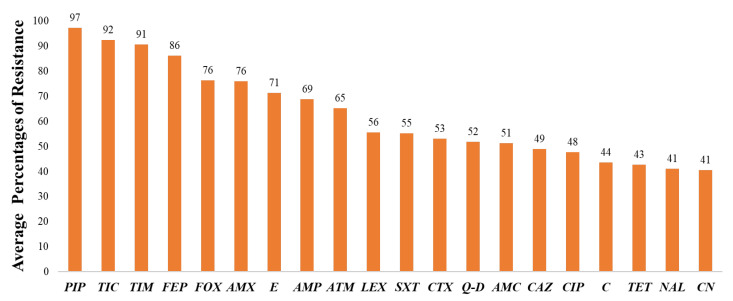
Major antibiotic resistance of Gram-negative bacteria in bats. Piperacillin = PIP, Ticarcillin = TIC, Ticarcillin-clavulanic acid = TIM, Cefepime = FEP, Cefoxitin = FOX, Amoxicillin = AMX, Erythromycin = E, AMP = Ampicillin, Aztreonam = ATM, Cephalexin = LEX, Cefotaxime = CTX, Quinupristin-dalfopristin = Q-D, Amoxicillin-Clavulanic acid = AMC, Ceftazidime = CAZ, Ciprofloxacin = CIP, Chloramphenicol = C, Tetracycline = TET, Nalidixic acid = NAL, Gentamicin = CN.

The average percentage of major antibiotic resistance in all Gram-negative bacteria obtained from bats is shown in Figure 3.

Recently, McDougall et al. reported in an Australian study that out of 39 *Klebsiella* spp. isolates, 30 showed different *bla*_SHV_ genes. However, phenotypic antimicrobial susceptibility testing confirmed some isolates (*n* = 13) exhibited intrinsic resistance to amoxicillin and ampicillin, but not ESBL activity [53]. Later in another study, they reported that *E. coli* (*n* = 13) isolated from bats represented ESBL-producers, as the isolates were positive for bla_CTX-M-27_ (7.6%) and bla_TEM-1B_ (84%) [70]. Obodoechi et al. also reported ESBL producing gene bla_CTX-M-15_ (5.7%) and bla_TEM_ (22.8%) [62] (Table S1).

#### 3.1.6. Other Gram-Negative Bacteria and Genes Responsible for Drug Resistance in Bats

In addition to *E. coli*, *Klebsiella,* and *Enterobacter*, several other Gram-negative pathogens such as *Citrobacter*, *Serratia,* and *Acinetobacter* associated genes responsible for AMR were reported [32,53,56]. In 1988, Graves et al. reported isolation of *Citrobacter* spp. from bats that were resistant to cephalothin (100%) [54]. Decades later in 2014, Obi et al. reported isolation of *Citrobacter freundii* that were highly sensitive to all drugs except sulfadimethoxin (28%) [56]. In a Brazilian study, Claudio et al. reported isolation of *Serratia marcescens*, *S. liquefaciens*, *A. baumannii*, and *Stenotrophomonas* spp. [32]. Out of 36 *S. marcesences* isolates, most were resistant to ampicillin (94%), amoxicillin-clavulanic acid (97%), and cephalexin (100%). All isolated *Sentrophomonous* spp. were resistant to ceftriaxone and imipenem [32]. Selvin et al. also reported ceftriaxone resistant *Escherichia furgusonii* [81]. Additionally, Sens-junior et al. reported that *S. liquefaciens* were resistant to amoxicillin (62.5%), amoxicillin-clavulanic acid (50%), and ampicillin (62.5%) [48].

Apart from ESBL genes, other antibiotic resistant genes were also detected in bats [31,63]. Nowakiewicz et al. confirmed the resistance profile of 38 *E. coli* isolates and further detected associated genes [31]. The study detected the *aph(3*′*)-iIa* gene responsible for kanamycin resistance, sulphonamide resistant genes *sul1* and *su2*, and gentamicin resistance determined by the presence of aac (3)-II, aac (3)-III isolates. All streptomycin-resistant isolates were characterized by the presence of the *strA* gene. Resistance to tetracycline was found by the presence of a single *tetA* gene, *tetB*, and both *tetA* and *tetB* genes. Genetic resistance to phenicols was confirmed by the presence of the *floR* gene in two isolates, the cm1A gene present in one isolate, and the cat gene in six isolates [31].

McDougall et al. reported that other than ESBL genes, *E. coli* isolates were positive for aminoglycoside resistance (APH(3″)-Ib + APH(6)-Id), trimethoprim resistance (*dfr*A14 + *sul2*), tetracycline resistance (*tetA*), kanamycin resistance (APH(3′)-Ia), and others [70]. Later, Nowakiewicz et al. published the AMR gene profile of *E. faecalis* and the isolates were positive for kanamycin resistance (aph(3′)-IIIa gene), high-level gentamicin-resistance (aac(6′)-Ie-aph (2″)-Ia), tetracycline resistant (*tetM*), and erythromycin resistance (*ermB*) [82] (Table S1).

Detection of streptomycin, tetracycline, sulfamethoxazole-trimethoprim, spectinomycin, and trimethoprim resistance genes were also found from *E. coli* isolated from bats [70]. Benavides et al., from Peru, also reported detection of 18 genes conferring aminoglycosides resistance at prevalence ranging from 3% (*aadB*) to 55% (*aadA1*) in multidrug-resistant *E. coli* [68] (Table S1).

### 3.2. Evidence of Antibiotic Resistant Gram-Positive Bacterial Pathogens in Bats

#### 3.2.1. *Staphylococcus aureus* and *Staphylococcus* spp.

AMR in Gram-positive bacteria remains a great challenge in infectious disease management [83]. Most studies focused on Gram-negative bacteria, as these are found as the predominant isolates from bat-originated specimens including fecal, cloacal, rectal, or guano samples [5,63,66]. Apart from Gram-negative bacteria, Gram-positive bacteria, especially *Staphylococcus* spp., also were isolated from bats [84,85,86].

An Australian study investigated semen, urethral, and preputial swabs from *Pteropus* bats and isolated *Streptococcus* and *Staphylococcus* as the predominate bacteria [87]. The most effective antibiotic against Gram-positive bacteria was penicillin, while the information of resistance against other broad-spectrum antibiotics was unclear. Two Nigerian studies, Akobi et al. and Olatimehin et al., reported isolation of 19.1% and 11.2% of *S. aureus* from fecal samples of the straw-colored fruit bat (*Eidolon helvum*) in 2012 [86] and 2018 [85], respectively. None of the studies observed MRSA prevalence, but both studies reported low levels of resistance against penicillin. *S. aureus* from the studies were found commonly colonized with ST1725 and ST1726 types of *S. aureus*. Akobi et al. (2012) reported no presence of Panton-Valentine leukocidin (PVL) virulent gene [86]. However, Olatimehin et al. (2018) detected PVL virulent gene in 78.6% of the isolates [85].

From Europe, reports of isolation of *Staphylococcus* spp. were found from both insectivorous and frugivorous bat species. In 2013, *Staphylococcus nepalensis* (*n* = 5) was identified from bat guano for the first time in Slovakia [84] and vancomycin resistance was reported in the same species in 2020 in the same country [65]. In addition to this species, other *Staphylococcus* species such as *S. xylosus*, *S. kloosii*, *S. nepalensis*, *S. simiae*, *S. aureus*, and *S. sciuri* were also reported in the United Kingdom [88] and Spain [89]. All *Staphylococcus* isolates in the Spanish [89] and 2013 Slovak [84] studies were resistant to erythromycin, and high streptomycin and tetracycline resistance also were reported. Fountain et al. reported 38.9% of *Staphylococcus* isolates to be amoxicillin resistant and 7.6% Coagulase negative *Staphylococcus* (CoNS) were cefoxitin resistant [88]. None of the *S. aureus* isolates showed phenotypic resistance to methicillin (screening agar) and none were found to carry *mecA* or *mecC*.

#### 3.2.2. Other Gram-Positive Organisms

**Figure 4:**
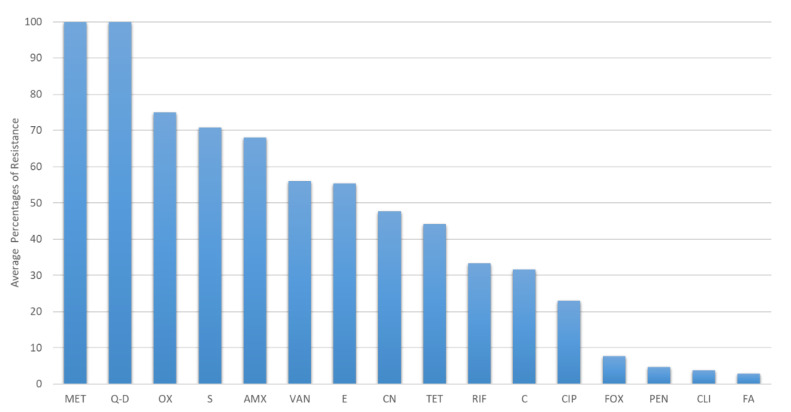
Major antibiotic resistance of gram-positive bacteria in bats. Methicillin = MET, Quinupristin-dalfopristin = Q-D, Oxacillin = OX, Amoxicillin = AMX, Streptomycin = S, Vancomycin = V, Erythromycin = E, Gentamicin = CN, Tetracycline = TET, Rifampicin = RIF, Chloramphenicol = C, Ciprofloxacin = CIP, Cefoxitin = FOX, Penicillin = PEN, Clindamycin = CLI, Fusidic acid = FA.

Other than CoNS and *S. aureus*, studies also reported other Gram-positive bacteria, such as *Kocuria, Bacillus*, and *Arthrobacter* [65,81,87]. Selvin et al. reported isolation of *Bacillus anthracis* from bats and the isolates were resistant to ciprofloxacin (25%), tetracycline (25%), and orfloxacin (75%) [81]. Gerbakova et al. reported isolation of *Arthrobacter* sp. resistant to chloramphenicol (50%) and vancomycin (50%) as well as *Kocuria* sp. resistant to chloramphenicol (18%) and vancomycin (18%) [65]. Recently, a Polish study by Nowakiewicz et al. reported isolation of *Enterococcus faecalis* from bat guano samples and the isolates were highly resistant to tetracycline (69.4%), streptomycin (41.7%), and kanamycin (38.9%) [82]. Another Spanish study also reported isolation of two *Enterococcus* isolates from bats’ rectal swabs; one out of two isolates were resistant to ciprofloxacin and erythromycin and both were resistant to quinupristin-dalfopristin [90]. The average percentage of antibiotic resistance in the Gram-positive bacteria obtained from bats is shown in Figure 4.

#### 3.2.3. Methicillin Resistant *Staphylococcus aureus* (MRSA)

MRSA is a widely found pathogen in hospital settings among Gram-positive *Staphylococcus*. Occurrence of MRSA also has been reported as a problem in veterinary facilities [91]. A 2008 study found one MRSA from two bat specimens (wound and gastrointestinal tract) [92]. Further molecular analysis was performed to understand the virulence properties of the isolates. SCC*mec* IV cassettes were found without panton-valentine leukocidine (PVL) genes in the bat MRSA. The detailed drug resistance profile of all bacterial isolates from bats (except *E. coli*) has been provided in the Supplementary Table S3 (Table S3).

## 4. Discussion

We have presented a detailed review of the AMR profile of bats’ bacterial commensals and pathogens that highlight their probable role in disseminating AMR in humans and the environment. Most studies in the field have focused on migratory birds as vectors for long-distance AMR dissemination, while the role of bats in disseminating AMR has been under investigated and is nascent in the literature [93]. Given the significant spatial and temporal heterogeneity in AMR distribution and the factors that affect its evolution, dissemination, and persistence, it is important to highlight that AMR must be viewed as an ecological problem. Thus, there is a significant interest worldwide in promoting a One Health perspective on AMR to enable a more accurate understanding of its ecosystem [94].

AMR in bat bacterial isolates (both Gram-positive and Gram-negative) were reported in parts of Asia (Indonesia, Malaysia, Japan, and India), North and South America (Brazil, Mexico, and Peru), Africa (Algeria, Nigeria, Gabon, Trinidad, and Republic of Congo), and Europe (Germany, Slovakia, Portugal, Slovenia, United Kingdom, Poland and Spain). Three studies from Brazil [32,48,95] and four studies from Nigeria [57,59,85,86] were very crucial in this review. These studies revealed a strong pattern of AMR profile of bat isolates as all three studies reported ampicillin, amoxicillin, amoxicillin-clavulanic acid, and cephalosporin’s resistance over the study period. Overall, these data showed bacterial isolates resistant to commonly used antimicrobials such as amoxicillin, amoxicillin-clavulanic acid, streptomycin, tetracycline, erythromycin, cefoxitin, and tetracycline. However, there was a record of high resistance to various other antimicrobials.

Drug resistance patterns have been observed in Gram-positive and Gram-negative isolates from various bat species around the world. In most of the studies, *E. coli* was the indicator organism that reported high resistance to clinically relevant antibiotics such as β lactams (ampicillin, amoxicillin, amoxicillin-clavulanic acid, and piperacillin), third-generation cephalosporins (ceftazidime and cefotaxime), aminoglycoside (streptomycin), tetracyclines, and quinolones (ciprofloxacin). Other than *E. coli*, all the Gram-negatives were also found highly resistant towards ampicillin and amoxicillin-clavulanic acid. Gentamicin resistance was checked by all the studies and all *E. coli* isolates were mostly sensitive towards gentamicin. Other reported organisms also were found sensitive toward gentamicin. Cefotaxime and ceftazidime resistance also were found by many studies, however, there were no trends observed in the level of resistance. Antimicrobials, especially fluoroquinolones, aminoglycosides, and third- and fourth-generation cephalosporins, are listed as critically important antimicrobials for human and veterinary use according to the World Health Organization (WHO) [96,97]. Resistance to common antibiotics by bats’ commensal bacterial flora is quite alarming and needs further evaluation. Supporting the statements of bats as a carrier of antimicrobial resistant bacteria, several published reports have shown resistance to β-lactams, cephalosporins, aminoglycosides, fluoroquinolones, and tetracycline in bacterial isolates from other wild mammals including wild boars, micro-mammals (wild rodents), and wild rabbits [4,6,98].

AMR exchange and transmission between wildlife, human, and domestic animals cannot be corroborated from the reports of phenotypic AMR only and as such genetic data are required to prove the existence of interfaces for resistance exchange and transmission. The collection of all antimicrobial resistance genes and their precursors in pathogenic and non-pathogenic bacteria as well as in antimicrobial producing-organisms is referred to as the antimicrobial resistome, a concept that has been advanced to serve as a framework for understanding the ecology of resistance on a global scale [99]. We have documented reports of genetic determinants of AMR in bats such as carbapenemase producing genes (*bla*_OXA_), ESBL genes (*bla*_TEM_, *bla*_CTX_, bla_SHV_), gentamicin (*aac (3)-II*, *aac (3)-III*), tetracycline (*tetA*, *tetB*), streptomycin (*strA*), and sulphamethoxazole (*sul1*, *sul2*). Previous studies also reported ESBL, AmpC β-lactamase, carbapenemase, colistin, tetracycline, chloramphenicol, and sulfonamide resistance genes in Enterobacteriaceae isolates of wildlife origin such as in wild birds and boars [15,73,74,100,101]. ESBL and carbapenemase producing pathogens conferring resistance to cephalosporins and carbapenem are currently major concerns for the treatment of human and veterinary illness worldwide and have been frequently reported in wildlife [6,13,102,103] Though reported in low numbers in bats and other wild mammals, development of resistance in such mechanisms is frightening.

Antibiotics released into the environment can apply selective pressure, promoting horizontal transfer of resistant genes in environmental bacterial communities and in wildlife bacterial flora [104,105]. Bats can also act as a carrier of antibiotic resistant genes and plasmids [57,63,106] and with their long distance flying and roaming capacities, they can broadly transmit those bacteria and genes to human and domesticated animal populations [48]. Still, the bat bacterial flora and their AMR profiles are poorly understood [31]. The acquisition of AMR microorganisms by bats could be due to AMR pollution that can occur through the exposure of wildlife to human food waste, wastewater treatment plants, and aquaculture operations with antimicrobial residues [107]. So far, it appears that the emergence of AMR occurs under selection and mostly by antibiotics; however, other components, such as heavy metals or biocides, may also play a role in the development of AMR. As a result, the presence of clinically relevant antimicrobial resistant genes and antibiotic-resistant bacteria in wild animals that are not getting antibiotics should be seen as a sign of AMR pollution [107,108,109].

As wild mammals, bats usually do not build specific shelters. Rather, they use natural caves and artificial habitats as resting or hibernating places [110]. Deforestation and food insecurity compel them to use urban and rural habitats, such as buildings and their ceilings, as roosting and foraging sites for breeding [111]. Habituating near human and domestic animals increases the likelihood of direct and indirect contact and sharing microflora. Anthropogenic activities, such as deforestation, hunting wild animals, and caving in areas where bats usually dwell, increases the likelihood of zoonotic infections associated with bats.

## 5. Conclusions

The present review provides an overview of available information on the antimicrobial susceptibility profile of bacteria isolated from bats. The origin of AMR in wildlife is currently a major global health concern due to identification of emerging resistant pathogens as well as the occurrence of frequent zoonotic pandemics, such as COVID-19. The current COVID-19 pandemic has also triggered a global AMR situation as many COVID positive patients were given antibiotics and were found to be colonized with highly resistant bacteria. There is a need to prioritize the concept of OH in order to improve the health of humans and animals, and there is a clear need for research of AMR from a wildlife or a zoonotic point of view. We observed that bats are a highly variable source of potential pathogenic and MDR bacteria, both Gram-positive and Gram-negative. Particularly, the prevalence of AMR genes (e.g., *CTX, TEM, SHV*) in bats is a major concern regarding AMR transmission dynamics in the wildlife–human–environment nexus. The rise of AMR during and following major pandemic events irrespective of causative pathogens requires strict vigilance of surveillance of zoonotic spillover events coupled with antibiotic susceptibility data. Extensive country- or region-specific OH studies to predict the direction and pattern of AMR in bats and wild animals need to be carried out for better planning, policy and stewardship program implementation.

## Data Availability

All data are available in the manuscript.

## Supplementary Materials

The following supporting information can be downloaded at: https://www.mdpi.com/article/10.3390/ijerph20010243/s1, Table S1: Summary of the included articles; Table S2: Antibiotic resistance profile of *E.coli* isolates in bats; Table S3: Antibiotic resistance profile of bacterial isolates (except *E.coli*) in bats.
